# Highly Sensitive Fluorescence Detection of Three Organophosphorus Pesticides Based on Highly Bright DNA-Templated Silver Nanoclusters

**DOI:** 10.3390/bios13050520

**Published:** 2023-05-05

**Authors:** Guowen Li, Xiufang Huang, Chifang Peng, Fengxia Sun

**Affiliations:** 1 State Key Laboratory of Food Science and Technology, Jiangnan University, Lihu Road 1800, Wuxi 214122, China; 2 School of Food Science and Technology, Jiangnan University, Lihu Road 1800, Wuxi 214122, China; 3 International Joint Laboratory on Food Safety, Jiangnan University, Lihu Road 1800, Wuxi 214122, China; 4 School of Food Science and Technology, Shihezi University, Shihezi 832000, China

**Keywords:** fluorescence sensor, DNA-templated silver nanoclusters, organophosphorus pesticides, fluorescence quenching

## Abstract

It is still challenging to achieve simultaneous and sensitive detection of multiple organophosphorus pesticides (OPs). Herein, we optimized the ssDNA templates for the synthesis of silver nanoclusters (Ag NCs). For the first time, we found that the fluorescence intensity of T base-extended DNA-templated Ag NCs was over three times higher than the original C-riched DNA-templated Ag NCs. Moreover, a “turn-off” fluorescence sensor based on the brightest DNA-Ag NCs was constructed for the sensitive detection of dimethoate, ethion and phorate. Under strong alkaline conditions, the P-S bonds in three pesticides were broken, and the corresponding hydrolysates were obtained. The sulfhydryl groups in the hydrolyzed products formed Ag-S bonds with the silver atoms on the surface of Ag NCs, which resulted in the aggregation of Ag NCs, following the fluorescence quenching. The fluorescence sensor showed that the linear ranges were 0.1–4 ng/mL for dimethoate with a limit of detection (LOD) of 0.05 ng/mL, 0.3–2 µg/mL for ethion with a LOD of 30 ng/mL, and 0.03–0.25 µg/mL for phorate with a LOD of 3 ng/mL. Moreover, the developed method was successfully applied to the detection of dimethoate, ethion and phorate in lake water samples, indicating a potential application in OP detection.

## 1. Introduction

Organophosphorus pesticides (OPs) have been used for agricultural pest control worldwide for over half a century, and they contribute 38% of the total global pesticide use [1]. However, OP residues cause serious contamination of agricultural products and environmental pollution due to improper use, and also bring about harm to human health through food intake [2]. Organophosphorus compounds are characterized by the presence of the thiophosphoryl functional groups (P=S). Excessive use of OPs causes long-term accumulation in the ecosystem, which is harmful to the environment and human beings, because it can inhibit the activity of acetylcholinesterase (AChE), which is a kind of acetylcholinesterase hydrolase (ATCh). If the activity of AChE is inhibited, organisms become overexcited, eventually leading to death [3,4].

It is of great significance to develop a rapid detection method for OP pesticides in environmental and food contamination. So far, the most common strategy of developing detection of OP has been mainly based on its inhibition of natural enzyme (AChE) activity [5,6]. However, AChE methods are generally used for OPs and carbamate pesticides [7]. Furthermore, Yan et al. utilized paraoxon as a representative of OPs to inhibit the activity of tyrosinase, resulting in the fluorescence recovery of egg-white-wrapped gold nanoclusters (Au NCs) and the detection of paraoxon [8]. Wu et al. used paraoxon to inhibit the activity of butyrylcholinesterase, recovering the fluorescence of carbon quantum dots (CQDs) which were quenched by gold nanoparticles (Au NPs) [9].

In addition, several works have employed hydrolytic enzymes to hydrolyze OPs, and have achieved the indirect detection of OPs through the detection of OP hydrolysates. For example, Su’s group employed OP hydrolase to hydrolyze parathion-methyl and produced p-nitrophenol [10]. This resulted in electron transfer (ET) from CdTe QDs to p-nitrophenol, thus quenching the CdTe QDs’ fluorescence and achieving the detection of parathion-methyl. However, it should be noted that environmental factors could greatly affect the activity of natural enzymes [11,12]. Limited works were also developed based on the chemical hydrolysis of OPs. Yi et al. hydrolyzed parathion-methyl to p-nitrophenol under alkaline conditions [13]. The hydrolysates entered the β-cyclodextrin (β-CD) cavity through host-guest recognition and quenched the fluorescence of β-CD-modified MoS_2_ QDs, thereby achieving the indirect detection of parathion-methyl. Liu et al. developed a highly sensitive fluorescence sensing system based on nitrogen and sulphur co-doped carbon dots for the indirect detection of methyl parathion with the aid of alkaline-induced hydrolysis [14]. Zhang et al. applied chemiluminescence induced by replacing methionine on the surface of Au-Fe_3_O_4_ dumbbell-like nanoparticles with parathion-methyl hydrolysates to detect parathion-methyl. The anti-interference effect in complex sample detection could be achieved by magnetic bead separation [15]. Compared with these methods, the fluorescence sensing method has the advantages of high sensitivity and simple operation.

Today, fluorescence quenching sensing based on the interaction between sulfhydryl groups and fluorescent nanoclusters is quite common. Most of these methods achieved detection based on intrinsic sulfhydryl groups [16]. Meanwhile, hydrolysis of OPs can also produce sulfhydryl groups [17]. However, there are few reports of sulfhydryl groups hydrolyzed from OPs applied in fluorescence sensing. Therefore, inspired by the quenching effect of sulfhydryl groups on fluorescent nanoclusters, we achieved fluorescence sensing of three OPs based on quenching fluorescence of DNA-templated silver nanoclusters (DNA-Ag NCs) using a pesticide hydrolysate. Herein, we developed highly sensitive fluorescence detection methods for the three OPs (dimethoate, ethion and phorate) based on the quenching of fluorescence of DNA-templated silver nanoclusters (DNA-Ag NCs) by the pesticide hydrolysate under alkaline conditions for the detection of three OPs. It was found that the rapid hydrolysis of dimethoate, ethion and phorate under strong alkaline conditions could be utilized to split the P-S bonds of three OPs and produce the sulfhydryl group. Then, an indirect sensitive response of the DNA-Ag NCs to the OPs was achieved through the sulfhydryl group bonding with Ag NCs and the formation of Ag-S bond. Additionally, the other common OPs could not interfere with the detection. The above method has the advantages of enzyme-free, simple operation, high sensitivity and good selectivity, thus, showing high application potential in OP detection.

## 2. Materials and Methods

### 2.1. Reagents and Instruments

The single-stranded DNA (ssDNA) shown in Table 1 was used in this study, and the oligonucleotide was synthesized by Shanghai Sangon Biotechnology Co. Ltd. (Shanghai, China). Silver nitrate (AgNO_3_) and sodium borohydride (NaBH_4_) were purchased from Shanghai Aladdin Biochemical Technology Co. Ltd. (Shanghai, China). Sodium hydroxide (NaOH) was provided by Sinopharm Chemical Reagent Co. Ltd. (Shanghai, China). The chemicals and reagents used were of analytical grade, and all pesticides were supplied by Shanghai Pesticide Research Institute. Ultrapure water used in this study was produced by Milli-Q Academic system (Millipore, Burlington, MA, USA; Resistivity: 18.2 MΩ·cm).

A JEOL JEM-2100 transmission electron microscope (Tokyo, Japan) was used for the transmission electron microscopy (TEM) measurement. The hydrodynamic size distribution was conducted on a dynamic light scattering (DLS) analyzer (Malvern Zetasizer Nano ZSE, London, UK). Fluorescence spectra were performed on a F97Pro spectrophotometer (Shanghai, China). The UV-Vis absorption spectrum was characterized on a UV-2802PCS UV–visible spectrophotometer (UNICO, Franksville, WI, USA).

### 2.2. Synthesis of DNA-Ag NCs

DNA-Ag NCs were synthesized by a one-step method according to previous reported methods with a slight modification [18,19], and the following provides the specific steps. Firstly, 16 µL of DNA solution (250 µM) was mixed with 166 µL of phosphate-buffered saline (PBS) (20 mM, pH 7.0). A volume of 6 µL of AgNO_3_ solution (4 mM) was then added to the mixture and incubated at 4 °C for 20 min. Subsequently, 12 µL of NaBH_4_ (2 mM) prepared with iced water was added. After being mixed with violent shaking, the mixture was then incubated in darkness at room temperature for 3 h. Finally, the prepared DNA-Ag NCs solution was stored at 4 °C until use.

### 2.3. Optimization of the Assay Conditions

To develop a highly sensitive and reproducible detection method for the detection of dimethoate, ethion and phorate, parameters such as NaOH concentration, hydrolysis temperature, hydrolysis time and the reaction time between DNA-Ag NCs and each pesticide were investigated. The NaOH solution was diluted to the concentrations of 1, 2, 3, 4 and 5 mM. The hydrolysis temperature of pesticides was examined at 20, 30, 50, 70 and 90 °C. The hydrolysis was performed at 0, 5, 10, 15 and 20 min for dimethoate, 0, 1, 3, 5, 10 and 15 min for ethion, and 0, 1, 3, 5 and 10 min for phorate, respectively. In addition, the reaction time between DNA-Ag NCs and each pesticide was also performed at 1, 5, 10, 15 and 20 min. The results of each assay are presented in terms of the fluorescence intensity of DNA-Ag NCs in the absence of pesticides (F0), the fluorescence intensity of DNA-Ag NCs in the presence of pesticides (F) and the ratio of the two (F/F_0_).

### 2.4. Pesticide Detection Procedure

The whole reaction process was divided into two steps: one was the hydrolysis of pesticides under alkaline conditions and the other was the reaction between hydrolysates and DNA-Ag NCs. In brief, 10 µL of pesticides (dimethoate, ethion and phorate) with different concentrations were mixed with 10 µL of sodium hydroxide solution (3 mM) and incubated for a certain time, respectively. Then, 60 µL of PBS (10 mM, pH 7.5) and 20 µL of DNA-Ag NCs solution (0.01×) were added and the reaction was carried out at room temperature. A volume of 95 µL of mixture was used to record the fluorescence intensity at 620 nm (excitation wavelength was 530 nm) by fluorescence spectrophotometry.

### 2.5. Detection of Dimethoate, Ethion and Phorate in Lake Water Samples

Lake water was collected from the local lake. After being filtered twice with a 0.22 μm microporous membrane, dimethoate (0.5, 2 and 4 ng/mL), ethion (0.5, 1 and 1.5 μg/mL), and phorate (0.05, 0.2 and 0.4 μg/mL) were added to lake water samples, respectively. The fluorescence analysis was followed by the above detection procedure, and the concentrations of pesticide residues in water samples were calculated using the standard curves. After the actual sample was prepared, the three pesticides with different concentrations were added to it, respectively, and the actual concentrations were detected by the standard curve. The recovery was calculated by the equation:

$$\mathrm{Recovery}=\frac{\mathrm{Deteced}\;\mathrm{concentration}\;}{\mathrm{Spiked}\;\mathrm{concentration}\;}\times 100\%$$

## 3. Results and Discussion

### 3.1. Sensing Strategy for the Detection of Three OPs

DNA can bind with silver ions through the N7 position of purine bases and the N3 position of pyrimidine bases with high affinity [20]. Therefore, the reduced silver atoms tend to aggregate inside the encapsulated spaces of nucleobases [21]. The luminescent properties of silver nanoclusters (Ag NCs) are closely related to their size [22]; when the size of Ag NCs approaches the Fermi wavelength of electrons, their energy structure breaks down into discrete levels which is similar to the molecule, rendering the Ag NCs with a good fluorescence quantum yield and resulting in a strong fluorescence intensity [23]. Based on this, the luminescent properties of silver nanoclusters can be adjusted by changing the DNA template sequence and structure.

**Figure 1:**
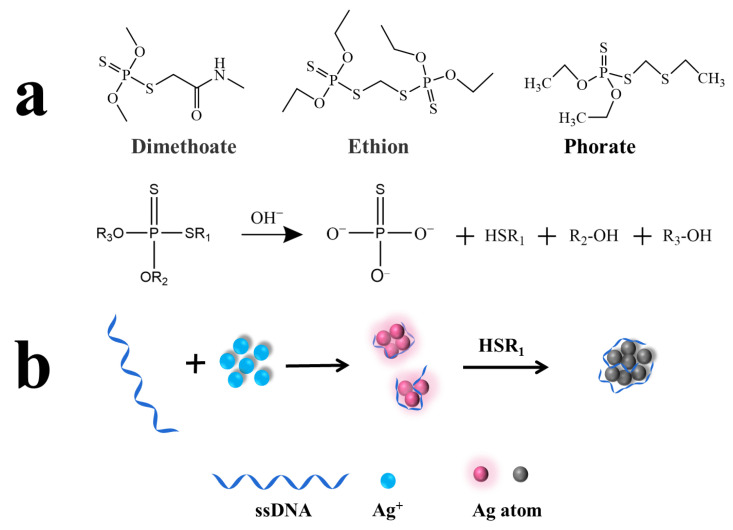
(**a**) The schematic illustration for the preparation of OP hydrolysates containing sulfhydryl groups; (**b**) the sensing principle of indirect detection based on DNA-Ag NCs.

In terms of the molecular structure, OPs can be divided into three categories: phosphate ester pesticides [(RO)_3_P=O], thiophosphate ester pesticides [(RO)_3_P=S] and thioester phosphate pesticides [(RO)_3_S-P=O(S)] [4]. According to previous reports, some OPs are easily hydrolyzed under alkaline conditions. Specifically, P-O or P-S bonds are broken to generate hydroxyl or sulfhydryl groups. For example, Lan et al. utilized the P-O bond fracture of parathion-methyl under alkaline conditions to produce p-nitrophenol, thus resulting in the inner-filter effect with N-doped carbon dots [24]. Under neutral conditions, P=S bonds coordinated with silver atoms on the surface of Ag NCs [25], which led to the weak fluorescence quenching of Ag NCs. However, in the strongly alkaline environment, P-S bonds were broken by pesticide hydrolysis and sulfhydryl groups were generated. Then, the sulfhydryl groups and the silver atoms on the surface of Ag NCs underwent a metal-sulfur coordination interaction, which greatly quenched the fluorescence of Ag NCs. As shown in Figure 1, the structures of dimethoate, ethion and phorate contain P-S bonds, which could hydrolyze under alkaline conditions and produce the sulfhydryl groups. Meanwhile, the highly bright DNA-Ag NCs were synthesized by sequestering AgNO_3_ with DNA and then reducing Ag^+^ to Ag^0^ clusters in the presence of NaBH_4_. Based on this, detection of the three OPs was achieved through the fluorescence quenching effect of the produced sulfhydryl groups on the DNA-Ag NCs which could be quantified by F/F_0_ (F and F_0_ represent fluorescence intensity in the presence and absence of Ops, respectively).

### 3.2. Characterization of DNA-Ag NCs

**Figure 2:**
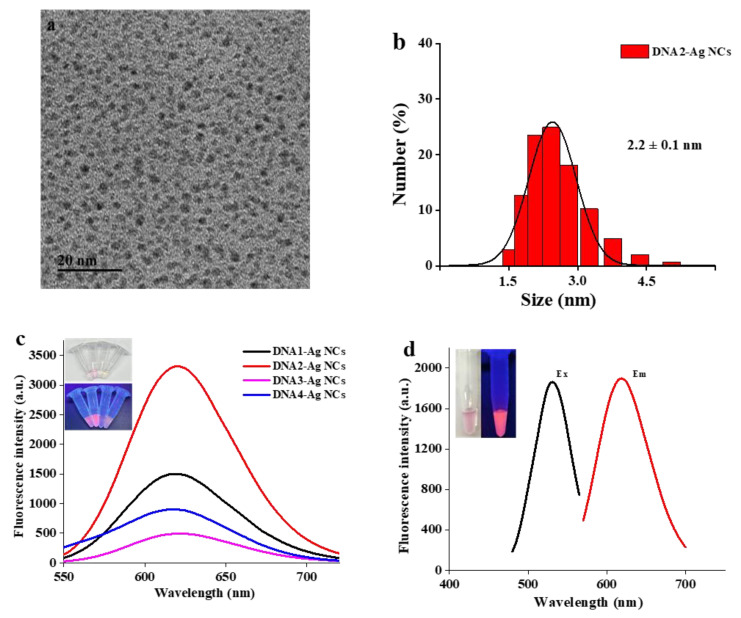
(**a**) TEM image of DNA2-Ag NCs. (**b**) DLS spectrum of DNA2-Ag NCs. (**c**) Fluorescence spectra of DNA-Ag NCs. The inserted digital images were DNA-Ag NCs illuminated with White (up) and UV (down) light. (**d**) Excitation and Emission spectra of DNA2-Ag NCs. The inserted digital images were DNA2-Ag NCs illuminated with white light (**left**) and UV light (**right**).

In the presence of ssDNA, DNA-Ag NCs were obtained by reducing AgNO_3_ with NaBH_4_. With the C-riched DNA1 template, we obtained Ag NCs with strong and stable fluorescence [26]. In order to obtain brighter Ag NCs, we designed another three ssDNA templates, which were obtained through extending several bases at two ends of the DNA1, and investigated their fluorescence characteristics. As shown in Figure 2b, we found that the fluorescence intensity of T-extended DNA-templated Ag NCs (DNA2-Ag NCs) was enhanced by more than double compared with DNA1-Ag NCs. However, the fluorescence intensities of A-extended (DNA3 Ag-NCs) and G-extended DNA-templated Ag NCs (DNA4-Ag NCs) were much lower than DNA1-Ag NCs. Werner et al. found that the red fluorescence of DNA-Ag NCs could be enhanced 500-fold when placed in proximity to guanine-rich DNA sequences [27]. Zhou et al. found that mixing ssDNA (T_20_)-templated Ag NCs and assistant DNA-Ag NCs (A_20_-C_55_-NC) could generate a new Ag NC luminescence center and promote fluorescence emission through the formation of paratactic parallel double strands after hybridization [28]. However, the fluorescence enhancement of DNA-Ag NCs in our work was obviously different from the above two typical phenomena of DNA-Ag NC enhancement. Considering that the change in the construction and microenvironment of the DNA template would alter the fluorescent characters of DNA-Ag NCs [28], we speculated that the enhanced fluorescence intensity of T-extended Ag NCs (DNA2-Ag NCs) was due to the fact that extended short T-bases could bind with the A-base existing in the C-riched DNA template, which slightly changed the microenvironment of the DNA template. To the best of our knowledgeable, this was the first obvious example of T-extended bases realizing the fluorescence enhancement of DNA-Ag NCs. Therefore, we selected the brightest DNA2-Ag NCs for further experiments.

In order to confirm the formation of DNA2-Ag NCs, the particle size of the synthesized nanoclusters was characterized. As shown in Figure 2a, TEM was employed to characterize the size and morphology of DNA-Ag NCs. The synthesized DNA2-Ag NCs displayed no aggregation and the average particle size was about 1.76 nm. As Figure 2b presents, the average particle size was about 2.2 nm. Compared with the TEM image, the particle size obtained by DLS is slightly larger, because DLS measures the hydrated particle size. It can be seen that the as-prepared DNA2-Ag NCs had uniform particle size distribution and no large aggregate particles were created. For fluorescence performance, the fluorescence spectra showed that the maximum emission wavelength of the DNA2-Ag NCs was 620 nm with an excitation wavelength of 530 nm (Figure 2d). Meanwhile, as shown in Figure S3 (Supplementary Materials), the fluorescence intensity of DNA2-Ag NCs decreased by about 10% in 6 months, indicating that the fluorescence intensity of the DNA2-Ag NCs was stable. Figure S1 presents X-ray photoelectron spectroscopy (XPS) data for the synthesized DNA-Ag NCs. As shown in Figure 1b, binding energy peaks at 368.1 eV and 374.1 eV ascribed to Ag 3d_5/2_ and Ag 3d_3/2_ were consistent with the standard reference XPS spectrum of Ag [29,30]. Meanwhile, the binding energy peaks at 402.8 eV in the XPS spectrum of N 1s also indicated binding of Ag and N [31].

### 3.3. Optimization of the Experimental Conditions

To achieve better sensing results of OPs, different concentration ratios of DNA, NaBH_4_ and silver ions were investigated for the synthesis of DNA-Ag NCs.

As shown in Figure S2, the fluorescence intensity of DNA-Ag NCs was gradually increased with the increase in silver ion concentration. The highest fluorescence intensity of DNA-Ag NCs was achieved at C_DNA_:C_Ag_^+^ = 1:6. Compared with the influence of DNA concentration, the influence of NaBH_4_ concentration was relatively small. The highest fluorescence intensity of DNA-Ag NCs was obtained at C_NaBH4_:C_Ag_^+^ = 1:6. Meanwhile, a higher concentration of NaBH_4_ caused a decrease in the fluorescence intensity of DNA-Ag NCs.

For the optimal fluorescence detection conditions, the experimental parameters including the concentration of NaOH, hydrolysis temperature, hydrolysis time, and the reaction time between targets and DNA2-Ag NCs were optimized.

**Figure 3:**
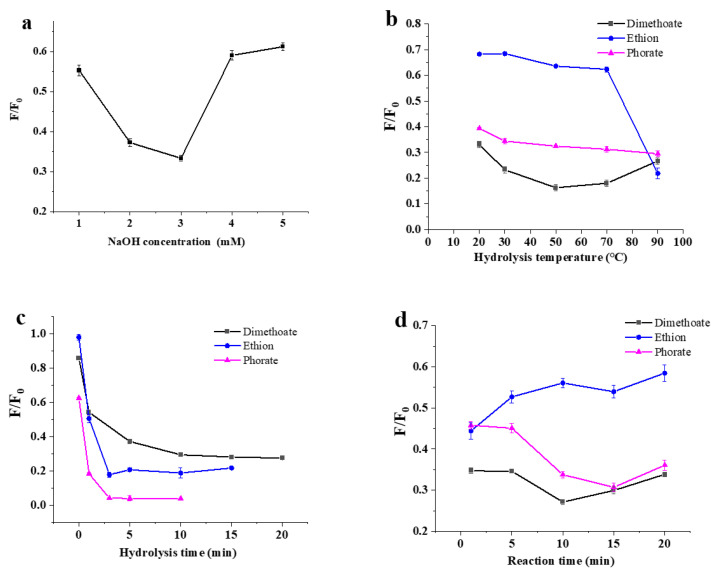
Optimization of the detection for three pesticides. (**a**) NaOH concentration, (**b**) pesticide hydrolysis temperature, (**c**) hydrolysis time, and (**d**) reaction time between pesticide and DNA2-Ag NCs.

The alkaline environment was provided by an NaOH solution for the hydrolysis of OPs. Pesticides were mixed with the NaOH solution at different concentrations (1, 2, 3, 4 and 5 mM) and hydrolyzed. As displayed in Figure 3a, 3 mM was the optimal NaOH concentration in the hydrolysis of dimethoate, ethion and phorate for the next fluorescent determination. The result was due to the fact that that the low concentration of NaOH could not cause sufficient hydrolysis, while too much NaOH affected the fluorescence stability of the DNA2-Ag NCs. The temperature was an important factor affecting the hydrolysis rate in the alkaline hydrolysis of OPs. As shown in Figure 3b, the F/F_0_ response to the hydrolysis of phorate was demonstrated by a slow decrease in the temperature fluctuation of 20–90 °C. As for dimethoate, an optimal temperature of 50 °C could be found, although only small F/F_0_ variations could be observed. Additionally, the F/F_0_ response to ethion hydrolysate was demonstrated by a sharp decrease in the heating curve from 70 to 90 °C. When the temperature was set at 90 °C, the hydrolysis of the three pesticides was adequate or nearly adequate. Thus, this temperature was selected for the next experiments.

From Figure 3c, it can be found that all the F/F_0_ values responding to the hydrolysis of the three pesticides sharply decreased within the initial 1–2 min, and were nearly stable after hydrolysis at 3 min. Thus, 3 min could be selected as the appropriate hydrolysis time. The reaction time of the pesticide hydrolysate with the Ag NCs was also investigated. As shown in Figure 3d, the F/F_0_ values decreased as the reaction progressed, indicating that the fluorescence of DNA-Ag NCs was quenched by three OPs. After a 5 min reaction time, the reaction was relatively stable. Thus, 5 min can be selected as the appropriate condition to conveniently facilitate the detection of the three OPs.

We must consider that although these three pesticides all contain P-S bonds in their structures, there are structural differences between them, which makes hydrolysis difficult and the optimal hydrolysis conditions different.

### 3.4. Fluorescence Assay of Dimethoate, Ethion and Phorate

**Figure 4:**
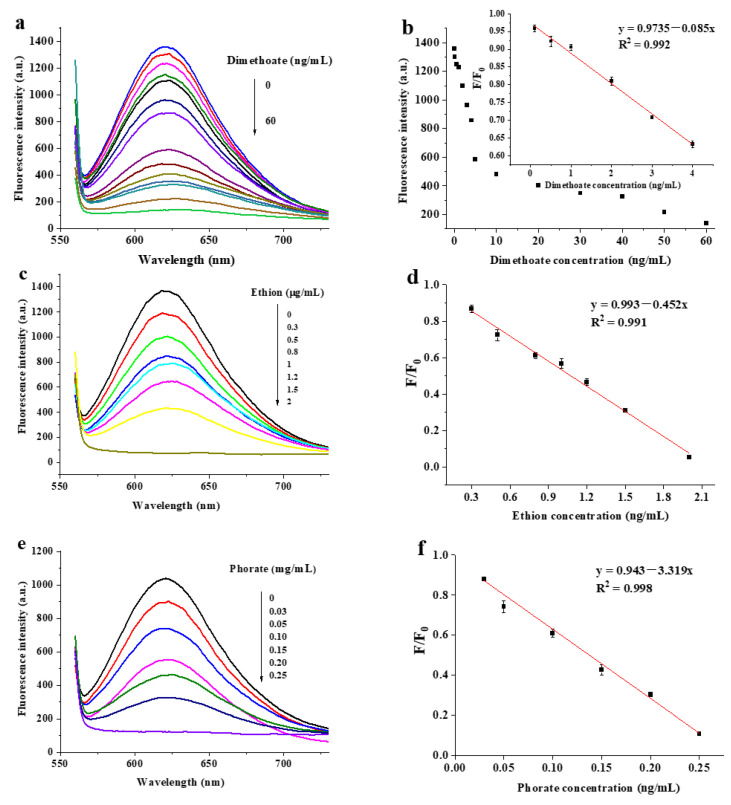
Fluorescence emission spectra of DNA2-Ag NCs at different concentrations of dimethoate (**a**), ethion (**c**) and phorate (**e**); the calibration plot of F/F_0_ versus concentration of dimethoate (**b**). Inset: corresponding linear ranges; corresponding calibration curves for ethion (**d**) and phorate (**f**) detection.

Different concentrations were added to study the sensitivity of the fluorescence sensor for the quantitative detection of the three pesticides under optimal conditions. The results are shown in Figure 4. The fluorescence intensity of DNA2-Ag NCs decreased accordingly with the increasing concentration of the three pesticides (Figure 4a,c,e). As shown in Figure 4b, the linear detection of F/F_0_ versus the dimethoate concentration was obtained in the range of 0.1–4 ng/mL. The linear regression equation for dimethoate was y = 0.9735 − 0.085x, and the correlation coefficient of R^2^ = 0.992. The LOD was calculated to be 0.05 ng/mL on the basis of 3σ/s (σ refers to the standard deviation of ten blank experiment values and s refers to the slope of the equation). When the ethion concentration was in the range of 0.3–2 µg/mL, the linear regression equation for ethion was y = 0.993 − 0.45x (R^2^ = 0.991) and the LOD was 0.03 µg/mL (Figure 4d). Figure 4f exhibits the plot of the F/F_0_ as a function of phorate concentration, and the regression curve was deduced to be y = 0.943 − 3.319x (R^2^ = 0.998). The linear range for phorate was 0.03–0.25 µg/mL and the LOD was 0.003 µg/mL.

**Figure 5:**
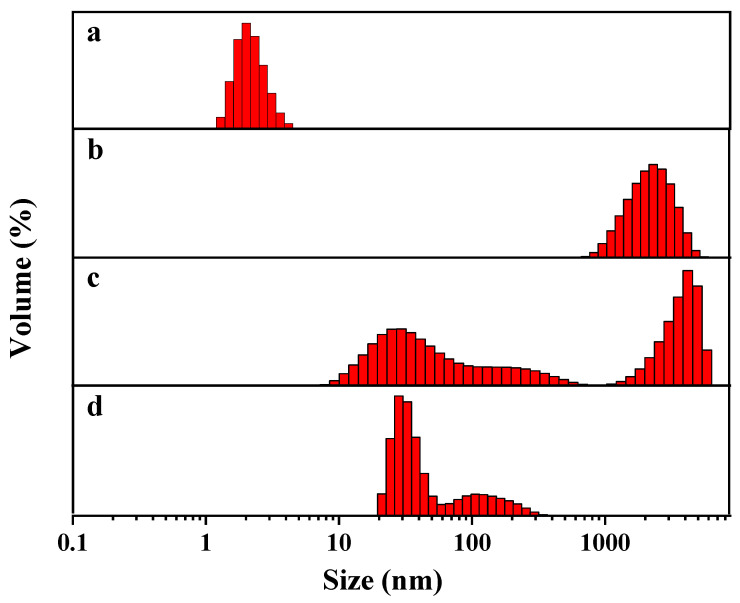
DLS images of DNA2-Ag NCs (**a**), DNA2-Ag NCs/Dimethoate (**b**), DNA2-Ag NCs/Ethion (**c**), and DNA2-Ag NCs/Phorate (**d**).

As mentioned above, the hydrolysis of dimethoate, ethion and phorate under alkaline conditions broke the P-S bonds and generated sulfhydryl groups. According to previous reports, the binding of sulfhydryl compounds to silver atoms on the surface of DNA-Ag NCs via Ag-S bonds resulted in the fluorescence quenching of nanoclusters [32]. We speculate that this was due to the aggregation of nanoclusters caused by the hydrolysates, which greatly quenched the fluorescence. The DLS images (Figure 5) indicate that the particle size of DNA2-Ag NCs was about 2 nm. When the hydrolysates of three pesticides were added, the particle size of the nanoclusters became larger and different degrees of aggregation appeared, which was consistent with our speculation.

In addition, this method does not require complex preparation and detection means and is simple to operate, showing more application prospects. There is an overview of its comparison with other previous reported sensors in Table 2, which indicates that the present method possesses remarkable advantages in terms of detection limit and multi-target detection versus reported publications. This sensor could be used for the sensitive analysis of dimethoate, ethion and phorate.

### 3.5. Selectivity Analysis

**Figure 6:**
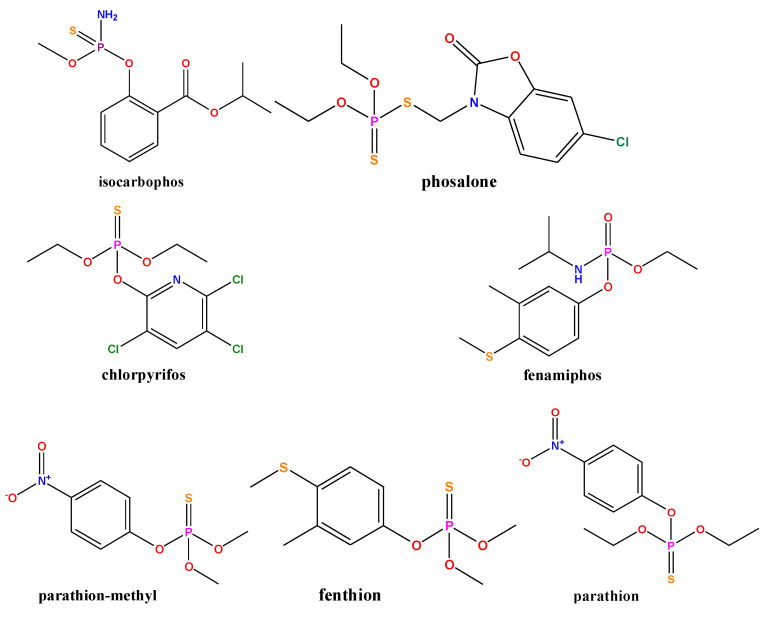
The chemical structures of seven OPs.

**Figure 7:**
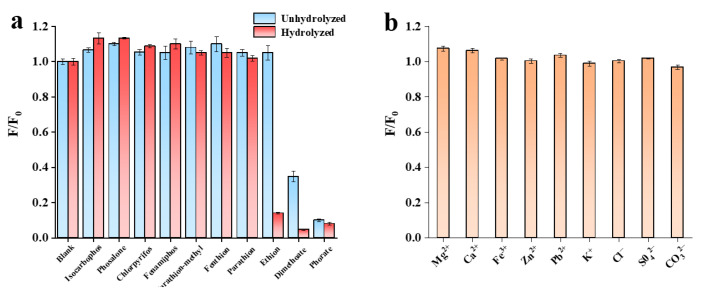
Selectivity of the fluorescence assay system toward dimethoate, ethion, and phorate at the same concentrations of 2 µg/mL, (**a**) against other pesticides at the same concentrations of 10 µg/mL, and (**b**) anion and cation at same concentration of 100 µM.

In order to evaluate the selectivity of the developed fluorescence sensor for target detection, another seven pesticides (isocarbophos, phosalone, chlorpyrifos, fenamiphos, parathion-methyl, fenthion, and parathion) were selected (Figure 6). Their structural formulae are shown in Figure 7a. Selectivity experiments were carried out under optimum conditions. The final concentrations of dimethoate, ethion and phorate were 2 µg/mL, five times lower than those of other interfering pesticides. As shown in Figure 7, these high concentrations of phosphate ester pesticides (fenamiphos), thiophosphate ester pesticides (isocarbophos, chlorpyrifos, parathion-methyl, fenthion, and parathion), and thioester phosphate pesticides (phosalone) did not respond to the DNA2-Ag NCs fluorescence system. Only three pesticide targets (dimethoate, ethion, and phorate) could cause fluorescence quenching. The response of DNA2-Ag NCs to dimethoate and ethion were greatly improved by hydrolysis under alkaline conditions. However, the hydrolysis of phorate only slightly enhanced its response. The reason could be due to the interaction between silver and sulfur also resulting in the breakage of the thioether bond in the phorate molecule, and the production of thiol [41]. Thus, the hydrolysis-based fluorescent detection had good selectivity for OPs containing P-bonds. We added some common anions and cations (100 μM) in the selective analysis experiment. As shown in Figure 7b, compared with the three OPs (2 µg/mL), a much higher concentration of interfering ions did not cause fluorescence quenching, indicating that the proposed method has good ion anti-interference ability.

### 3.6. Detection of Dimethoate, Ethion and Phorate in Real Samples

To verify the practical application of the DNA2-Ag NC fluorescence sensor for the lake water samples spiked with different concentrations of dimethoate, ethion and phorate were tested with the above fluorescent method. The results in Table 3 showed that the average recovery ranges for dimethoate, ethion, and phorate detection were 89–105%, 97–110% and 90–110%, and the relative standard deviations were 2.1–8%, 3.5–7.2% and 4.0–9.5%, respectively. These results indicate that the proposed fluorescence sensor is a promising application prospect for dimethoate, ethion and phorate detection in real water samples with high accuracy and reliability.

## 4. Conclusions

In this work, we obtained the Ag NCs with a stronger fluorescence intensity by extending T bases at two ends of an original C-riched DNA template. Based on this, a fluorescence sensor was successfully designed for dimethoate, ethion and phorate detection. In this sensor, DNA-Ag NCs combined with the hydrolysates of these pesticides under alkaline conditions and formed non-fluorescent polymers by Ag-S bonds, which could effectively quench the fluorescence emission of DNA-Ag NCs. Under optimum conditions, the LODs of dimethoate, ethion and phorate reached 0.05 ng/mL, 30 ng/mL and 3 ng/mL, respectively. The developed fluorescence sensor showed good sensitivity and selectivity of OPs containing P-S bonds, and had satisfactory recovery levels in lake water samples. Hence, our proposed sensor is a potential tool that can achieve the sensitive detection of residues of three OPs in real samples with a low cost and simple operation.

## Figures and Tables

**Table 1:** Sequence of five single-stranded DNAs.

DNA	Sequence 5′-3′
DNA 1	ACCCGAACCTGGGCTACCACCCTTAATCCCC
DNA 2	TTACCCGAACCTGGGCTACCACCCTTAATCCCCTT
DNA 3	AAACCCGAACCTGGGCTACCACCCTTAATCCCCAA
DNA 4	GGACCCGAACCTGGGCTACCACCCTTAATCCCCGG

**Table 2:** Comparison of proposed method and other reported methods for the detection of OPs.

Method	Target	Linearity (ng/mL)	LOD (ng/mL)	Reference
Colorimetry	Dimethoate	10–400	4.7	[33]
Colorimetry	Dimethoate	20–160	14	[34]
Colorimetry	Phorate	0.5–5000	0.1667	[35]
Gas chromatography	Dimethoate Phorate	10–1000 10–1000	1.8 0.8	[36]
Electrochemistry	Dimethoate	0.01–300	0.0063	[37]
Fluorescence	Dimethoate	20–3200	6	[38]
Fluorescence	Dimethoate	5–150	2.1	[39]
Fluorescence	Dimethoate	6–200	2.24	[40]
Fluorescence	Dimethoate Ethion Phorate	0.1–4 300–2000 30–250	0.05 30 3	This study

**Table 3:** Determination of the three pesticides in lake water samples (*n* = 3).

Pesticide	Spiked (ng/mL)	Found (ng/mL)	Recovery (%)	RSD (%)
Dimethoate	0.5	0.45	89	4.0
	2.0	1.90	95	2.1
	4.0	4.20	105	8.0
Ethion	500	510	102	7.2
	1000	970	97	3.5
	1500	1650	110	4.0
Phorate	50	48	96	6.2
	200	180	90	4.0
	400	440	110	9.0

## Data Availability

All data generated or analyzed during this study are included in the published article.

## Supplementary Materials

The following supporting information can be downloaded at: https://www.mdpi.com/article/10.3390/bios13050520/s1, Figure S1: (a) The whole XPS spectrum (b) Ag 3d (c) N1s XPS spectrum of DNA2-Ag NCs; Figure S2: Optimization of synthesis conditions of DNA2-Ag NCs; Figure S3: The stability of DNA2-Ag NCs.
