# Supplementary material for: Highly Sensitive Fluorescence Detection of Three Organophosphorus Pesticides Based on Highly Bright DNA-Templated Silver Nanoclusters
Source: Biosensors (Basel). 2023 May 5;13(5):520. doi: 10.3390/bios13050520 (PMC10216281; doi:10.3390/bios13050520)
Supplement: Supplementary file 1 [file biosensors-13-00520-s001.zip › biosensors-2337890-supplementary.pdf]

Supporting Information

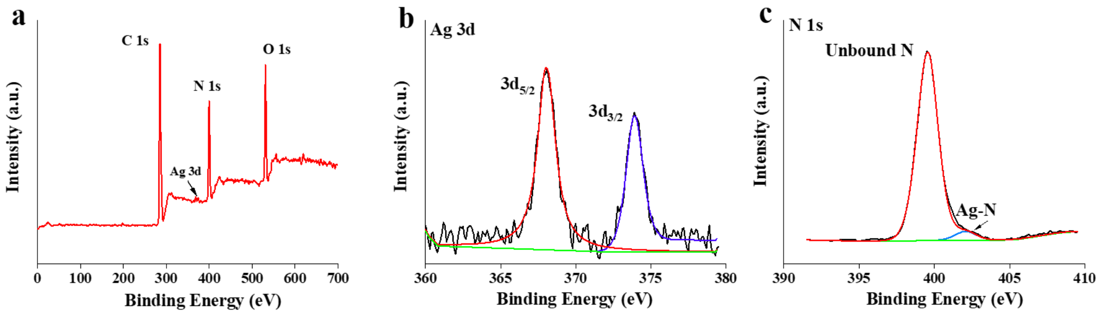

Figure S1. (a) The whole XPS spectrum (b) Ag 3d (c) N1s XPS spectrum of DNA2-Ag NCs

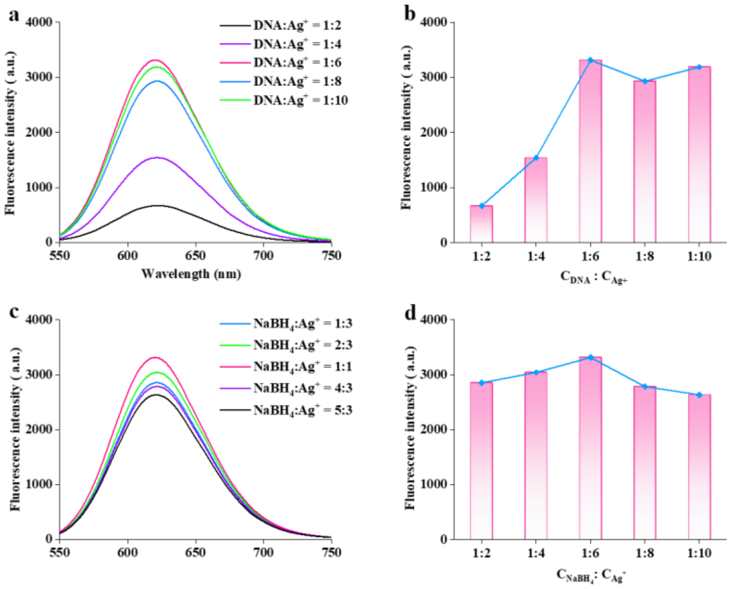

Figure S2. Optimization of synthesis conditions of DNA2-Ag NCs. Fluorescence spectrum of optimization on (a) DNA: Ag^+ (c) $NaBH_4$: Ag^+. The histogram figure of optimization on (b) DNA: Ag^+ (d) $NaBH_4$: Ag^+

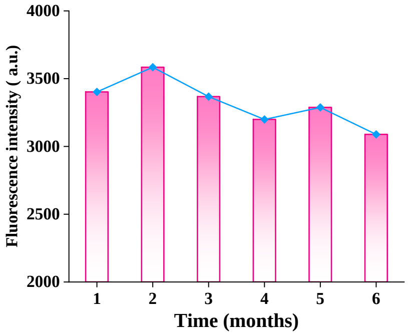

Figure S3. The stability of DNA2-Ag NCs
